# PHIP hyperpolarized [1-^13^C]pyruvate and [1-^13^C]acetate esters via PH-INEPT polarization transfer monitored by ^13^C NMR and MRI

**DOI:** 10.1038/s41598-021-85136-2

**Published:** 2021-03-11

**Authors:** Alexandra Svyatova, Vitaly P. Kozinenko, Nikita V. Chukanov, Dudari B. Burueva, Eduard Y. Chekmenev, Yu-Wen Chen, Dennis W. Hwang, Kirill V. Kovtunov, Igor V. Koptyug

**Affiliations:** 1 International Tomography Center SB RAS, 3A Institutskaya St., Novosibirsk, Russia 630090; 2 Novosibirsk State University, 2 Pirogova St., Novosibirsk, Russia 630090; 3 Institute of Cytology and Genetics SB RAS, 10 Ac. Lavrentieva Ave., Novosibirsk, Russia 630090; 4 Department of Chemistry, Wayne State University, Detroit, MI 48201 USA; 5 Karmanos Cancer Institute, Wayne State University, Detroit, MI 48201 USA; 6 Integrative Biosciences, Wayne State University, Detroit, MI 48201 USA; 7 Russian Academy of Sciences, Moscow, Russia 119991; 8 Institute of Biomedical Sciences, Academia Sinica, Taipei, 115 Taiwan (Republic of China)

**Keywords:** Solution-state NMR, Chemical physics, Chemical physics

## Abstract

Parahydrogen-induced polarization of ^13^C nuclei by side-arm hydrogenation (PHIP-SAH) for [1-^13^C]acetate and [1-^13^C]pyruvate esters with application of PH-INEPT-type pulse sequences for ^1^H to ^13^C polarization transfer is reported, and its efficiency is compared with that of polarization transfer based on magnetic field cycling (MFC). The pulse-sequence transfer approach may have its merits in some applications because the entire hyperpolarization procedure is implemented directly in an NMR or MRI instrument, whereas MFC requires a controlled field variation at low magnetic fields. Optimization of the PH-INEPT-type transfer sequences resulted in ^13^C polarization values of 0.66 ± 0.04% and 0.19 ± 0.02% for allyl [1-^13^C]pyruvate and ethyl [1-^13^C]acetate, respectively, which is lower than the corresponding polarization levels obtained with MFC for ^1^H to ^13^C polarization transfer (3.95 ± 0.05% and 0.65 ± 0.05% for allyl [1-^13^C]pyruvate and ethyl [1-^13^C]acetate, respectively). Nevertheless, a significant ^13^C NMR signal enhancement with respect to thermal polarization allowed us to perform ^13^C MR imaging of both biologically relevant hyperpolarized molecules which can be used to produce useful contrast agents for the in vivo imaging applications.

## Introduction

^13^C labeling of carboxyl groups of key biomolecules such as acetate and pyruvate can be employed for in vivo magnetic resonance (MR) studies of metabolism. Complementary to ^13^C-labeled glucose widely employed in metabolic studies^1^, acetate is used in the MR studies of metabolism in brain^2–4^, liver^5^, and muscles^6,7^, in particular, to address the tricarboxylic acid cycle, which is the main cycle in energy homeostasis in animals^2,8,9^ and humans^10^. Moreover, with ^13^C-labeled acetate it is possible to gain more accurate information on glia-specific processes because it is metabolized in glia and not in neurons^3^. Another key molecule is pyruvate, which is important for the energy-generating pathways in mammals^11^. The differences in the metabolic conversion of pyruvate to lactate help to differentiate normal cells from malignant tumor cells. For example, the study by Golman et al. demonstrated the possibility to detect [1-^13^C]pyruvate-derived metabolites in the abdominal tumor cells in mice^11^. The studies of prostate cancer in humans using [1-^13^C]pyruvate were also performed safely^12^. The studies that followed expand the range of [1-^13^C]pyruvate MR imaging applications further^13–18^.

Therefore, the applications of pyruvate and acetate ^13^C MR are driven by the central role of these metabolites in putative metabolic pathways. Moreover, the use of ^13^C nucleus provides two practical advantages over ^1^H MR: an enhanced chemical shift dispersion and the lack of background signal^19^. However, ^13^C MR spectroscopy (MRS) and imaging (MRI) face additional sensitivity challenges because of the lower ^13^C gyromagnetic ratio compared to ^1^H nuclei. Recently, ^13^C NMR hyperpolarization techniques have been introduced to boost MR detection sensitivity by up to 4–5 orders of magnitude. As a result, hyperpolarization methods paved the way for in vivo ^13^C MRI and MRS applications by taking advantage of the enhanced sensitivity. Importantly, ^13^C T_1_ relaxation times are substantially longer compared to those of protons^20^, and therefore ^13^C sites can act as excellent carriers of hyperpolarization. For example, under in vivo conditions the T_1_ relaxation time of lactate methyl protons is only 1.7 s, while the carboxyl carbon T_1_ time is 30 s for [1-^13^C]pyruvate^21^. This provides a sufficient time window for the preparation, administration and circulation of ^13^C-hyperpolarized metabolites and for monitoring their metabolic pathways.

Currently, dissolution dynamic nuclear polarization (d-DNP)^22^ is the leading hyperpolarization technique for in vivo applications, with the majority of all MRI studies utilizing hyperpolarized (HP) ^13^C-labeled pyruvate and acetate as well as other metabolites being performed using d-DNP^23^. However, the associated high costs^24^ of the d-DNP instrumentation and its operation is a major obstacle for a much wider use of this technique. Alternatively, the technique termed parahydrogen-induced polarization (PHIP)^25^ is an emerging method for hyperpolarizing ^13^C nuclei^26^. One of its variations, PHIP-SAH (PHIP using side-arm hydrogenation)^27^, is an elegant approach in which a side-arm fragment of an ester is hydrogenated via pairwise parahydrogen (p-H_2_) addition while the carboxylic moiety (*e.g*., pyruvate or acetate) remains unchanged. Reineri et al. produced HP acetate by hydrogenation of vinyl acetate to ethyl acetate, followed by ^1^H to ^13^C polarization transfer via magnetic field cycling and subsequent hydrolysis of ethyl acetate with sodium hydroxide^28^. Subsequent studies extended this approach to lactate^29^ and pyruvate^30,31^. Consequently, PHIP-SAH technique rekindled the interest in p-H_2_-based approaches for the production of HP contrast agents for in vivo MR metabolic studies.

Upon hydrogenation of a precursor molecule with p-H_2_, hyperpolarization is initially produced for ^1^H nuclei and thus needs to be transferred to a ^13^C nucleus. There are two general approaches to this task: magnetic field cycling (MFC)^19,32^ and application of radio-frequency (RF) pulse sequences^32–36^. Both approaches have their merits. In particular, the pulsed RF approach allows one to perform polarization transfer directly in the MRI scanner used for ^13^C MR imaging^37^. The generation of HP compounds directly in the detection area excludes polarization losses caused by T_1_ relaxation and magnetic field variation during the transfer of a HP compound to the imaging instrument. Indeed, previous studies^37,38^ demonstrated the possibility to perform polarization transfer step and ex vivo and in vivo MRI at the same location.

Many RF pulse sequences are suitable candidates for the polarization transfer step. Among these, PH-INEPT is one of the most widely used pulse sequences for polarization transfer from protons to heteronuclei^39^. This pulse sequence transforms the singlet spin order derived from p-H_2_ into an anti-phase magnetization of a heteronucleus. An extension of this method, PH-INEPT-PLUS, allows one to refocus the anti-phase magnetization into the in-phase magnetization^40^, which is advantageous for imaging experiments.

In this work, biologically relevant [1-^13^C]pyruvate and [1-^13^C]acetate esters were hyperpolarized via the PHIP-SAH technique, followed by polarization transfer from p-H_2_-derived protons to ^13^C nuclei by two different approaches, the optimized PH-INEPT-type pulse sequences and the MFC protocol, for their direct comparison under identical conditions. Based on these results, ^13^C MRI of HP [1-^13^C]pyruvate and [1-^13^C]acetate esters was performed successfully.

## Results

**Figure 1:**
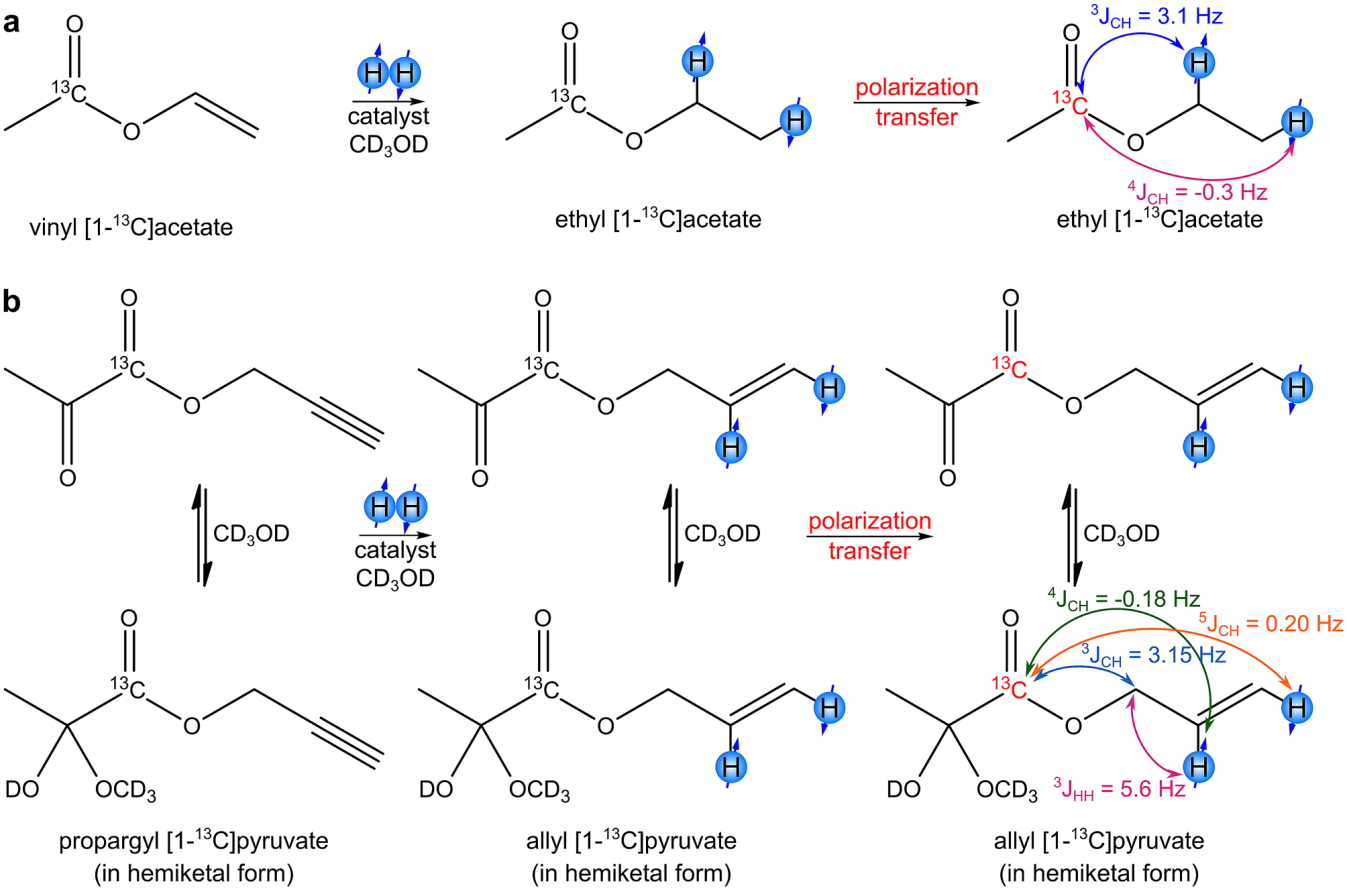
The reaction scheme of pairwise addition of parahydrogen to vinyl [1-^13^C]acetate (**a**) and propargyl [1-^13^C]pyruvate (**b**) in a homogeneous hydrogenation process. The corresponding J-couplings in the hydrogenation product molecules are shown with the arrows. In the case of allyl pyruvate, J-couplings are provided for the hemiketal form, which is prevalent in a CD_3_OD solution.

Vinyl [1-^13^C]acetate and propargyl [1-^13^C]pyruvate^41,42^ (Fig. 1) were used as precursors for the production of corresponding HP molecules via hydrogenation of the unsaturated side-arm moieties to obtain the highest ^13^C polarization levels^43^. Two catalysts were used in the homogeneous hydrogenation: [Rh(dppb)(NBD)]BF_4_ (dppb = 1,4-bis(diphenylphosphino)butane; NBD = norbornadiene) prepared from commercially available [Rh(NBD)_2_]BF_4_ (Strem Chemicals, 96%) and dppb ligand (Sigma-Aldrich, 98%) in a 1:1 ratio, and commercially available [Rh(dppb)(COD)]BF_4_ (COD = 1,5-cyclooctadiene, Sigma-Aldrich, 98%). CD_3_OD was used as the solvent. Further details are provided in Supplementary Table S1.

### Ethyl [1-^13^C]acetate

**Figure 2:**
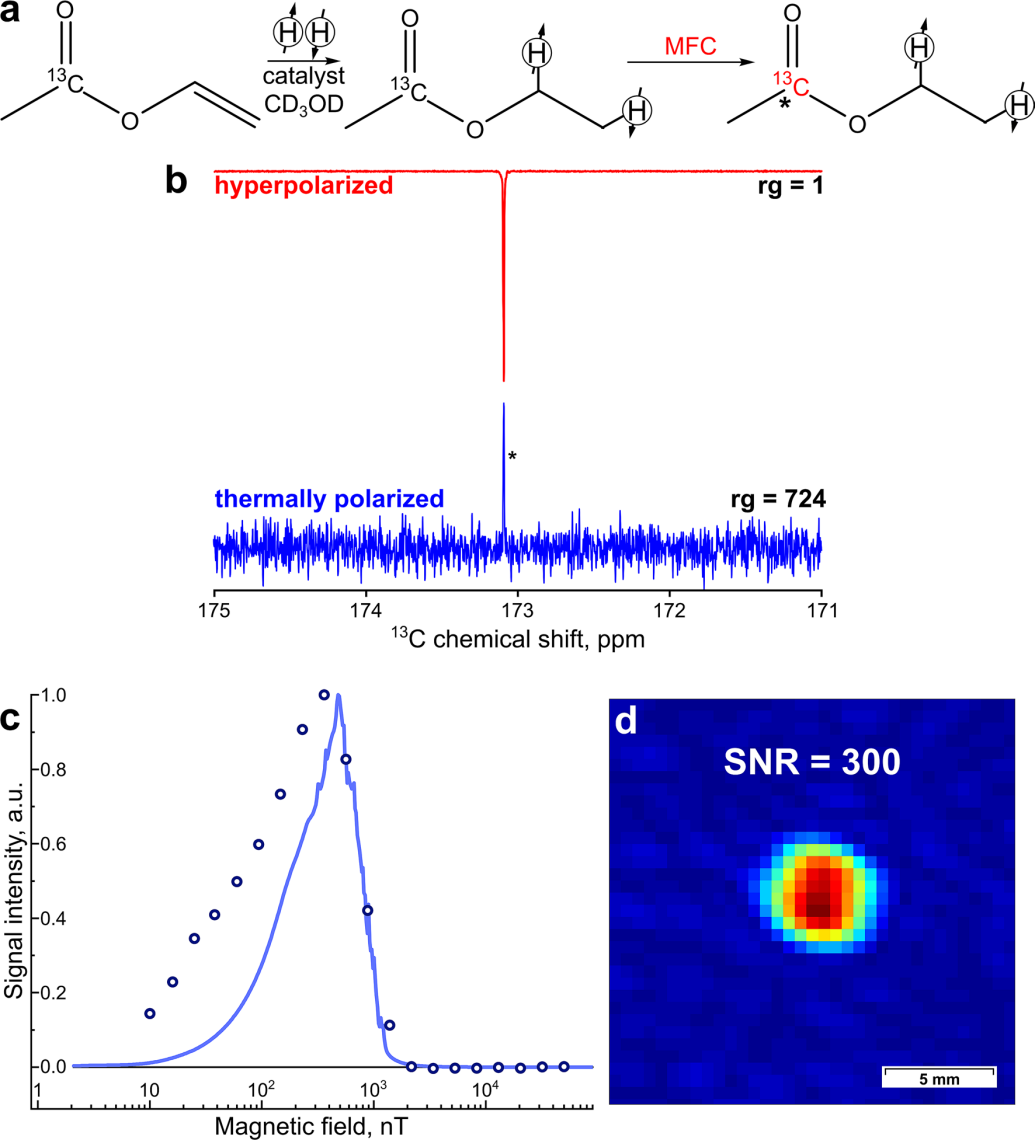
(**a**) The reaction scheme of vinyl acetate hydrogenation. (**b**) The ^13^C NMR spectrum acquired after ^13^C hyperpolarization of ethyl [1-^13^C]acetate using MFC with a 400 nT magnetic field in the shield (red line) with the receiver gain (rg) value of 1, and the corresponding thermal ^13^C NMR spectrum acquired after relaxation of hyperpolarization (blue line) with rg = 724. The carboxyl carbon in acetate is marked with *. For both spectra, proton decoupling was used during signal acquisition (**c**) The dependence of P(^13^C) of ethyl acetate with natural abundance of ^13^C isotope on magnetic field inside the shield in MFC experiments. The solid blue line corresponds to the numerical calculation; the dots correspond to the experimental data. (**d**) The ^13^C MR image acquired at 9.4 T after ^13^C hyperpolarization of ethyl [1-^13^C]acetate using MFC with a 400 nT magnetic field in the shield. FOV = 3.07 × 3.07 cm^2^. The initial 64 × 16 matrix was zero-filled to 64 × 64. The resulting images were zoomed to eliminate the uninformative parts. The spatial resolution after zero-filling is 0.48 × 0.48 mm^2^/pixel.

First, to estimate an optimal magnetic field for polarization transfer, MFC experiments were performed by changing the field inside the magnetic shield mounted on top of a spectrometer using an automated MFC setup described elsewhere^44^. Experimental details and numerical calculation procedures can be found in Supplementary Information (SI). The substrates with natural isotopic abundance were used in these experiments (Fig. 2). All data is presented normalized to the maximum value of the experimental field dependence. The resulting field dependence of the intensity of the enhanced carboxyl ^13^C NMR signal of ethyl acetate on the polarization transfer field shows a single maximum at 400 nT (Fig. 2c). As clearly seen in Fig. 2c, experimental data is in a general agreement with numerical calculations.

Next, MFC experiments were repeated using ^13^C-labeled vinyl acetate. The maximum observed ^13^C polarization P(^13^C) was 0.65 ± 0.05%, which corresponds to 1100-fold signal enhancement (SE) at 7 T (Fig. 2b). This allowed us to obtain a ^13^C image with the signal-to-noise ratio (SNR) of 300 (Fig. 2d).

**Figure 3:**
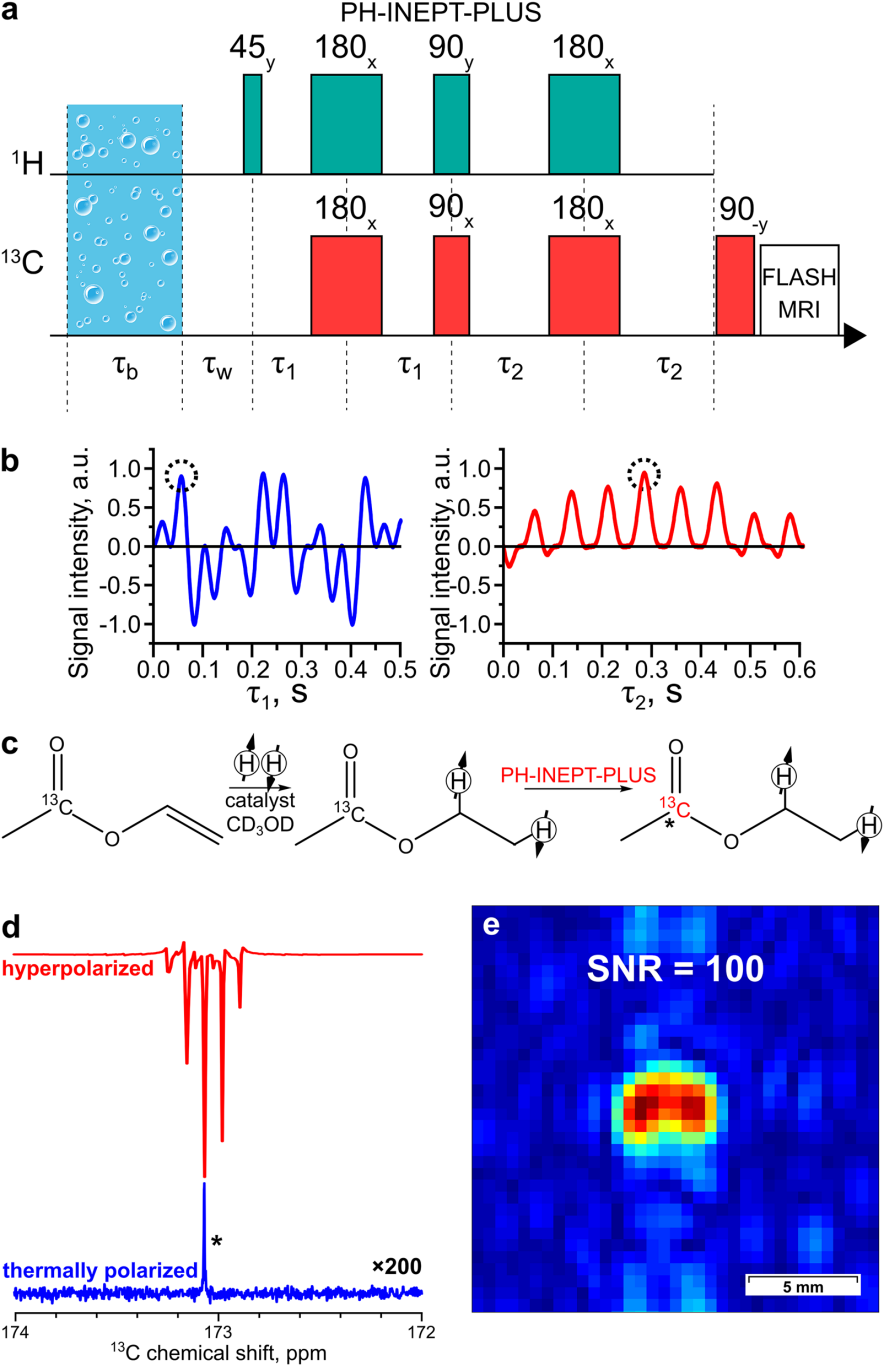
(**a**) The PH-INEPT-PLUS pulse sequence used to transfer polarization from ^1^H to ^13^C nuclei. (**b**) Numerical simulation of the PH-INEPT-PLUS sequence efficiency as a function of delays τ_1_ and τ_2_. The X axis represents the duration of the corresponding delay, whilst the Y axis shows the normalized polarization of the target carbon atom. Dotted black circles mark the delay values τ_1_ = 0.057 s and τ_2_ = 0.284 s that were used for polarization transfer in the experiments with ethyl [1-^13^C]acetate. (**c**) The reaction scheme of vinyl acetate hydrogenation. (**d**) The ^13^C NMR spectrum acquired after ^13^C hyperpolarization of ethyl [1-^13^C]acetate using the PH-INEPT-PLUS pulse sequence (red line), and the corresponding thermal ^13^C NMR spectrum acquired after relaxation of hyperpolarization (blue line, the intensity is multiplied by a factor of 200). The carboxyl carbon in acetate is marked with *. Proton decoupling was used during signal acquisition of the thermally polarized spectrum because otherwise the intensity of the signal was too low. (**e**) The ^13^C MR image acquired at 9.4 T after ^13^C hyperpolarization of ethyl [1-^13^C]acetate using the PH-INEPT-PLUS pulse sequence. The image FOV, matrix size, and resolution are the same as in Fig. 2d.

For RF polarization transfer based on the INEPT pulse sequence (Fig. 3), spin echo delays τ_1_ and τ_2_ were numerically optimized to maximize the calculated ^13^C polarization levels P(^13^C) (the detailed information on the calculation of optimal delays in the PH-INEPT-PLUS pulse sequence can be found in “Methods” section). Experimental implementation was based on the PH-INEPT-PLUS pulse sequence (Fig. 3a) with τ_1_ = 0.057 s and τ_2_ = 0.284 s (Fig. 3b). This optimization allowed us to obtain 0.19 ± 0.02% ^13^C polarization and SNR = 100 for a ^13^C MR image (Fig. 3e). In both cases, the initial concentration of vinyl [1-^13^C]acetate was 80 mM and its conversion to ethyl [1-^13^C]acetate was 40%; therefore, the concentration of HP ethyl [1-^13^C]acetate was 32 mM.

### ***Allyl [1-***^***13***^***C]pyruvate***

**Figure 4:**
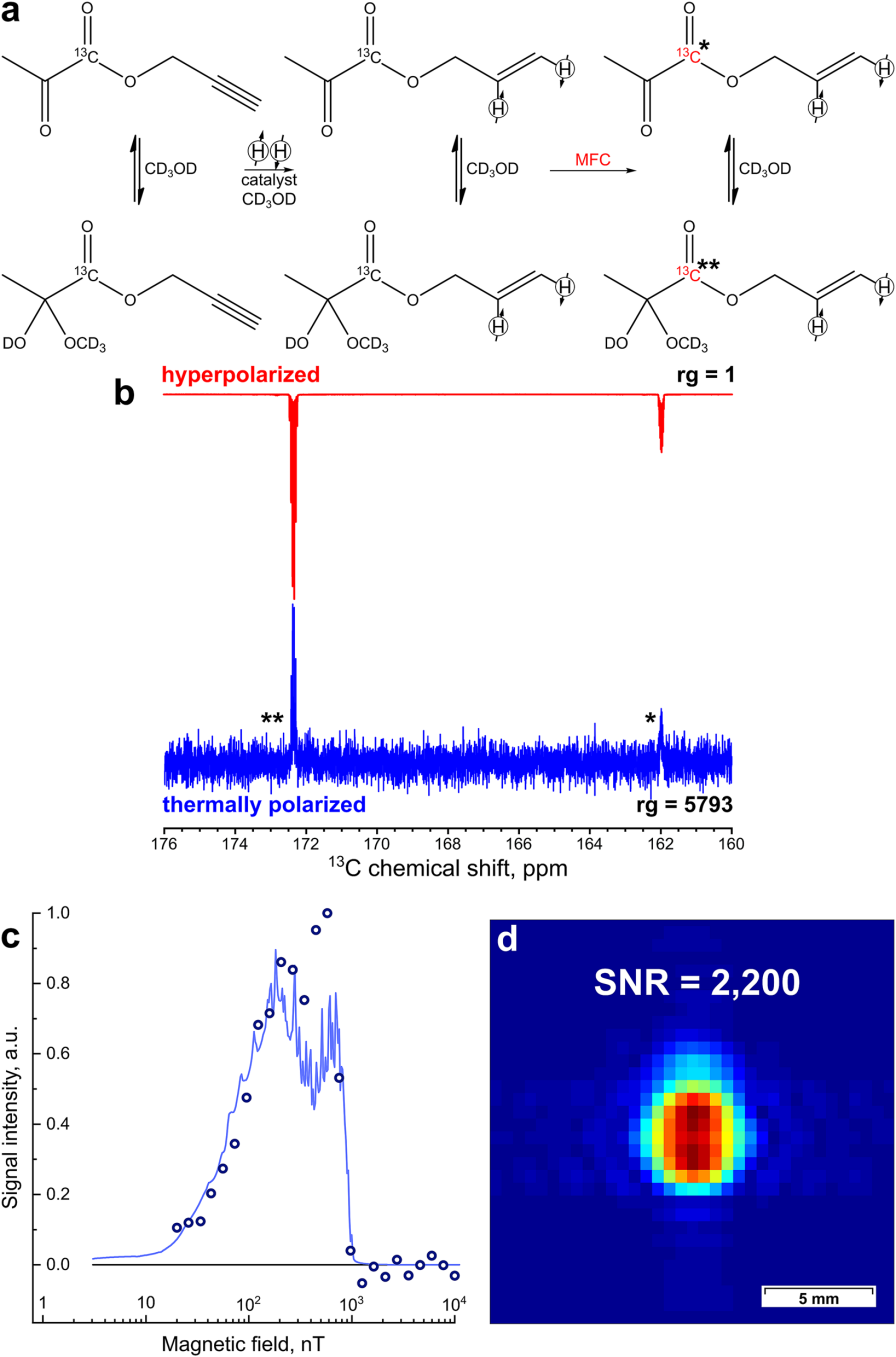
(**a**) The reaction scheme of propargyl pyruvate hydrogenation. (**b**) The ^13^C NMR spectrum acquired after ^13^C hyperpolarization of allyl [1-^13^C]pyruvate using MFC with a 200 nT magnetic field and rg = 1 (red line), and the corresponding thermal ^13^C NMR spectrum acquired after relaxation of hyperpolarization with rg = 5793 (blue line). The carboxyl carbon in pyruvate is marked with * in the keto form and with ** in the hemiketal form. (**c**) The dependence of P(^13^C) of allyl pyruvate in the hemiketal form with natural ^13^C abundance on magnetic field in MFC experiments. The solid blue line corresponds to the numerical calculation; the dots correspond to the experimental data. (**d**) The ^13^C MR image acquired at 9.4 T after ^13^C hyperpolarization of allyl [1-^13^C]pyruvate using MFC with a 200 nT magnetic field in the shield. The image FOV, matrix size, and resolution are the same as in Fig. 2d.

For allyl pyruvate (Fig. 4), the field dependence has a more complex appearance due to a large number of different J-couplings in the product molecule. The calculated optimal field for the polarization transfer is 200 nT (Fig. 4c), however, the experimental maximum is located at a slightly higher field. It should be noted that any such simulation is necessarily semi-quantitative at best. The fine details of the calculated curve may depend significantly on numerous parameters which are very difficult to match precisely between the experiment and simulation. Therefore, the simulation results provide the general guidance, while the optimum parameters are further refined in the experiments.

**Figure 5:**
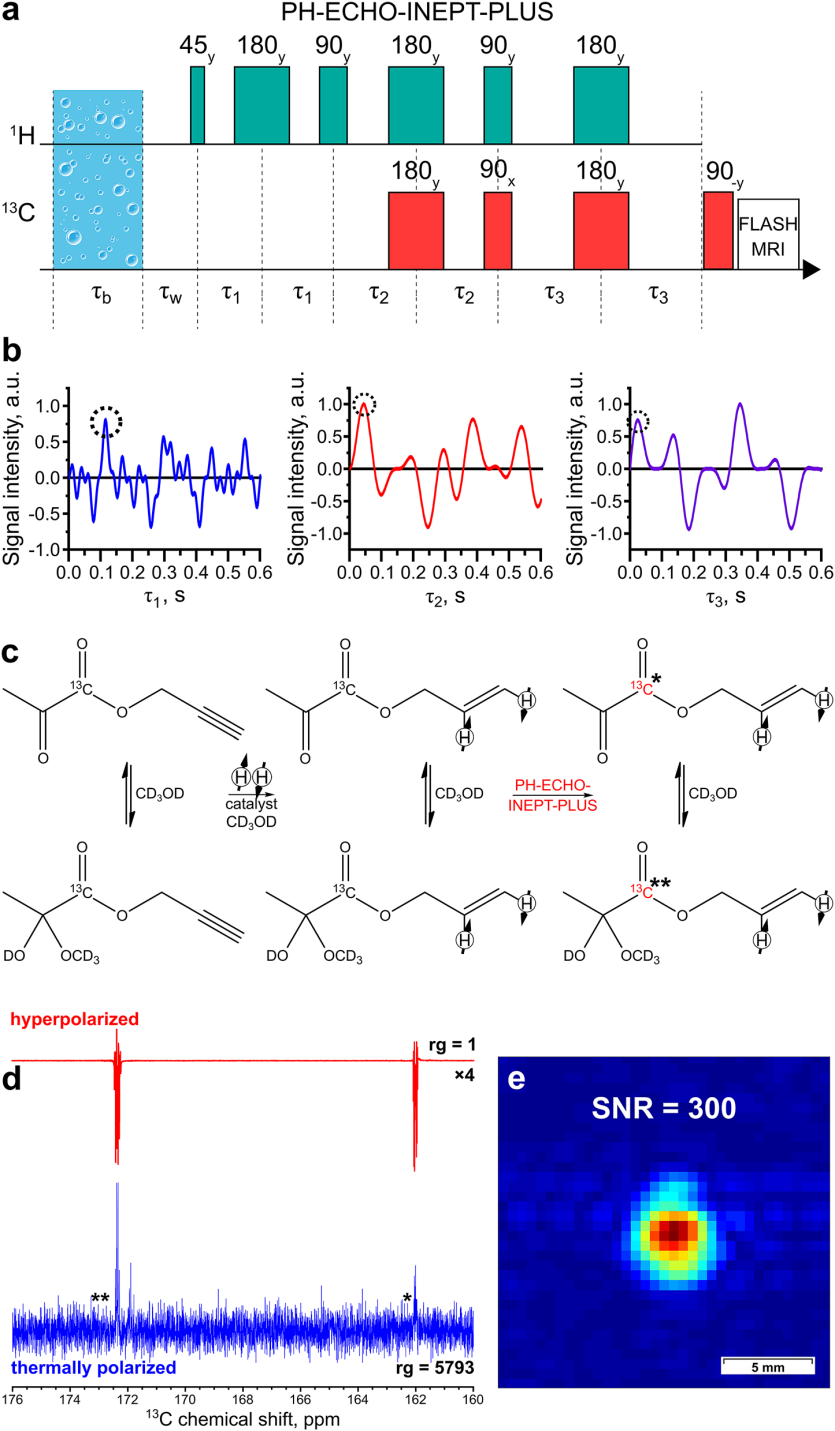
(**a**) The PH-ECHO-INEPT-PLUS pulse sequence used to transfer polarization from ^1^H to ^13^C. (**b**) Numerical simulation of PH-ECHO-INEPT-PLUS efficiency as a function of τ_1_, τ_2_, and τ_3_ delays. The X axis represents the duration of the corresponding delay, whilst the Y axis shows the normalized polarization of the target carbon. Dotted black circles mark the delay values τ_1_ = 0.115 s, τ_2_ = 0.045 s, and τ_3_ = 0.035 s that were used in the polarization transfer experiments with allyl [1-^13^C]pyruvate. (**c**) The reaction scheme of propargyl [1-^13^C]pyruvate hydrogenation. (**d**) The ^13^C NMR spectrum acquired after ^13^C hyperpolarization of allyl [1-^13^C]pyruvate using the PH-ECHO-INEPT-PLUS pulse sequence with rg = 1 (red line; multiplied by a factor of 4) and the corresponding thermal ^13^C NMR spectrum acquired after relaxation of hyperpolarization with rg = 5793(blue line). The carboxyl carbon in pyruvate is marked with * in the keto form and with ** in the hemiketal form. (**e**) The ^13^C MR image acquired at 9.4 T after ^13^C hyperpolarization of allyl [1-^13^C]pyruvate using the PH-ECHO-INEPT-PLUS pulse sequence. The image FOV, matrix size, and resolution are the same as in Fig. 2d.

MFC experiments were then performed using propargyl [1-^13^C]pyruvate. The maximum observed P(^13^C) was 3.95 ± 0.05% (SE = 6900 at 7 T), which allowed us to obtain a ^13^C image with a SNR of 2200 (Fig. 4d). The polarization level is presented for the hemiketal form because it is prevalent in a CD_3_OD solution. When vinyl moiety is used as a side-arm, two p-H_2_-derived protons are added to the carbon–carbon double bond at three to four bonds away from the carboxyl carbon, while with propargyl moiety the two protons are added at four to five bonds away from the target ^13^C nucleus. Therefore, implementing the PH-INEPT-PLUS pulse sequence to hyperpolarize carboxyl ^13^C atom in allyl pyruvate is problematic because of extremely small J-coupling constants between the p-H_2_-derived protons and the ^13^C nucleus (Fig. 1). In this case, numerical optimization yields τ_1_ = 0.786 s and τ_2_ = 2.046 s, which in practice would result in a poor performance because of proton polarization decay induced by T_2_ relaxation and diffusion of polarized molecules during polarization transfer. To deal with this issue, we added an additional spin-echo block to the PH-INEPT-PLUS sequence (Fig. 5a). The resulting pulse sequence, denoted as PH-ECHO-INEPT-PLUS, allows one to transfer proton polarization through an intermediate group of nuclei. In allyl [1-^13^C]pyruvate the role of these nuclei is fulfilled by protons in the methylene group (-CH_2_-) of the allyl fragment, which have reasonable J-couplings to both p-H_2_-derived protons and the target carbon (Fig. 1). Optimization of the delays was performed in the same way as for PH-INEPT-PLUS, giving the following values: τ_1_ = 0.115 s, τ_2_ = 0.045 s and τ_3_ = 0.035 s (Fig. 5b). Using an optimized PH-ECHO-INEPT-PLUS pulse sequence (Fig. 5c), we observed a maximum P(^13^C) of 0.66 ± 0.04% (Fig. 5d), which allowed us to acquire a ^13^C MR image with the SNR of 300 (Fig. 5e). The conversion of propargyl [1-^13^C]pyruvate to allyl [1-^13^C]pyruvate was 80–90%, i.e., higher than that obtained for [1-^13^C]acetate esters (40%). The initial concentration of propargyl [1-^13^C]pyruvate was 80 mM; therefore, the concentration of HP allyl [1-^13^C]pyruvate was 64–72 mM.

### ^13^C NMR/MRI on Bruker BioSpec 7 T instrument

During the experiments on an animal MRI scanner, the parahydrogen fraction of only 50% was provided by a home-built parahydrogen generator operating at liquid nitrogen temperature. The concentrations of the substrates were increased from 0.08 M to 0.8 M for vinyl [1-^13^C]acetate and to 0.18 M for propargyl [1-^13^C]pyruvate. The additional reason to increase the concentration of the to-be-polarized substrate is a longer time for sample transfer to the animal imaging scanner compared to conventional vertical bore instruments. The resulting ^13^C MR images have SNR = 70 for ethyl [1-^13^C]acetate (Supplementary Fig. S2) and SNR = 560 for allyl [1-^13^C]pyruvate (Supplementary Fig. S3) despite the fact that the slice thickness was 45 mm for the [1-^13^C]acetate ester and 10 mm for the [1-^13^C]pyruvate ester. In should be noted that ester cleavage, administration of the HP substrate and its metabolic conversion in the in vivo experiments all lead to the dilution of HP substances and relaxation-induced hyperpolarization losses. Nevertheless, the SNR values presented for the MR images acquired on the solution in an NMR tube can serve as a useful reference to give an idea about SNR achievable during future in vivo studies.

## Discussion

### MFC experiments

Polarization transfer in the PHIP-SAH approach using MFC has been reported previously^28,43,45,46^. For example, in the study of Cavallari and coauthors^46^, two concentric μ-metal cylinders were used to shield the laboratory’s magnetic field. The carboxyl carbon-13 polarization of 2.3% was observed. Subsequent studies with an optimized MFC profile showed 4.8% ^13^C polarization for allyl lactate^29^ and 5–6% for allyl pyruvate^27,30^. In another study^48^, the polarization levels obtained for ethyl [1-^13^C]acetate and allyl [1-^13^C]pyruvate were 4.4% and 5.4%, respectively. The difference between the values obtained in our work and the other studies can be explained by different polarization transfer protocols. In our experiments, p-H_2_ was bubbled through the solution in the μ-metal shield and then transferred to the high magnetic field of the spectrometer (see “Methods” section for more details). In the other studies, the sample was hydrogenated at the Earth’s magnetic field with subsequent transfer to the μ-metal shield and then to the spectrometer. In addition, in one study^48^ the polarization for allyl [1-^13^C]pyruvate was evaluated using the sum of the signal intensities of both pyruvate forms, while we used only the hemiketal form for this. We also note that the polarization vs. magnetic field dependences obtained in this work are different from those reported earlier^28,43,45,46^. In the MFC procedure employed in this work, the maximum polarization corresponds to the evolution of the spin system in the magnetic field which puts it the level anticrosing (LAC)^47^ region, whereas an adiabatic sweep through the LAC is more efficient if started at a much lower magnetic field. Thus, evolution of the spin system in our work occurred for a substantial period of time (hydrogenation for 20 s in the experiments) in the LAC region^47^ instead of an adiabatic passage through this region employed in a more conventional MFC polarization transfer procedure. While the latter can be somewhat more efficient, the MFC procedure used in our work was deemed advantageous as its experimental optimization was carried out on an automated setup capable of achieving a non-adiabatic field jump. This facilitates the comparison of the experimental results with numerical modeling of the field dependence because it avoids the variability of the conventional adiabatic MFC performed manually, and in addition simplifies the mathematical modeling of the process.

### INEPT-based experiments

The efficiency of polarization transfer in the RF-based methods depends on the J-couplings between the p-H_2_-derived protons and the heteronucleus of interest in the product molecule, with generally lower efficiency of polarization transfer for smaller heteronuclear J-coupling values^48^. Thus, a straightforward implementation of PH-INEPT for polarization transfer in acetate and pyruvate derivatives is encumbered because J-coupling values between the carboxyl ^13^C nucleus and the p-H_2_-derived protons in the hydrogenated side-arm moiety are relatively small (Fig. 1). The efficient spin order transfer to heteronuclei via relayed INEPT chains (ESOTHERIC)^49^ method allows one to surpass this problem by using a set of sequential INEPT steps on a specially designed precursor molecule isotopically labeled with ^13^C and ^2^H nuclei.

For example, in the study of Korchak et al.^50^ ^13^C polarization of ethyl acetate-d_6_ obtained using ESOTHERIC pulse sequence was ca. 59% at the natural abundance of the ^13^C isotope. Better efficiency than the one achieved in this study can be explained by a higher conversion of vinyl acetate to ethyl acetate and a higher pH_2_ concentration than in our study. In addition, the substrate molecule was deuterated. In another study, Dagys et al.^51^ showed 7.6% of ^13^C polarization during hydrogenation of 3-butyn-2-ol-d_5_, also using the ESOTHERIC pulse sequence and a deuterated substrate. The pulse sequence contained three INEPT blocks and transferred hyperpolarization from parahydrogen protons to a relayed proton, with further transfer of hyperpolarization to the heteronucleus. This method modification was called PR-SAH (proton-relayed SAH). The authors also demonstrated the hydrolysis and phase separation, which led to 4.3% ^13^C hyperpolarization of [1-^13^C]pyruvate in aqueous solution. While these studies achieved better results, the need for such a specific labeling is a clear disadvantage as it drastically increases the synthetic complexity and the cost of the PHIP precursor^52–54^. Moreover, the introduction of deuterium atoms may have deleterious effects on polarization transfer^55^. In addition, in the case of singly ^13^C-labeled molecules, it is often preferable to make the RF pulse sequence as simple as possible to reduce the undesired coherent evolution in a complex spin system. Thus, PH-INEPT-PLUS and its modifications remain more relevant for polarization transfer in molecules hyperpolarized by PHIP-SAH than the complex ESOTHERIC pulse sequence.

### Potential future improvements

In the future experiments, we expect to achieve an improved catalytic conversion using high-pressure spray injection hyperpolarizer equipped with RF pulsing and in situ detection capability^56,57^, and additionally improve the polarization yields using a larger p-H_2_ enrichment fraction^58,59^ and an optimized sample transfer procedure. For in vivo applications, the use of aqueous medium is required instead of toxic CD_3_OD. There are several studies devoted to the observation of PHIP effects using aqueous media^60,61^. Therefore, the future improvements mentioned above and the use of aqueous medium with water-soluble catalysts or the aqueous extraction procedures will certainly provide better results and will be compatible with in vivo MRI.

## Conclusions

In this work, two different approaches to polarize ^13^C nuclei by the PHIP-SAH method in molecules possessing an acetate or a pyruvate moiety are reported and compared. The first one is based on the magnetic field cycling, which is the most popular approach used in the previous PHIP-SAH studies. The resulting ^13^C polarization levels were 3.95 ± 0.05% for [1-^13^C]pyruvate and 0.65 ± 0.05% for [1-^13^C]acetate. The second approach is based on the INEPT-type pulse sequences to transfer polarization from ^1^H to ^13^C in a high magnetic field. It significantly simplifies the in vitro imaging procedure because no sample shuttling is required. The observed polarization levels were 0.66 ± 0.04% for [1-^13^C]pyruvate and 0.19 ± 0.02% for [1-^13^C]acetate. The achieved polarization levels allowed us to perform ^13^C MRI on a standard research scanner at 9.4 T and on a 7 T animal imaging scanner. The results demonstrate the possibility to observe polarization levels that are high enough for ^13^C MRI on two biologically relevant molecules using MFC and PH-INEPT-type pulse sequences in combination with the PHIP-SAH approach.

## Methods

### Experimental details

In the experiments, an unsaturated substrate and a catalyst were dissolved in methanol-d_4_. Vinyl acetate with natural ^13^C abundance (Sigma-Aldrich) was used as received; propargyl pyruvate with natural ^13^C abundance and ^13^C-labeled vinyl [1-^13^C]acetate and propargyl [1-^13^C]pyruvate were synthesized according to the procedures reported elsewhere^42^. In most experiments, hydrogen gas was enriched with para-isomer using Bruker Parahydrogen Generator BPHG-90 operating at 43 K, resulting in 85% p-H_2_ fraction. The magnetic field cycling experiments were carried out using a three-layer μ-metal shield (Magnetic Shield Corp., USA), with an additional solenoid inside it to adjust the magnetic field in the shield. NMR spectra were acquired on a 7 T Bruker AV 300 NMR spectrometer. Acquisition of the NMR spectra during MFC experiments was started 0.3 s after the placement of the sample into the NMR spectrometer using a 90-degree pulse without any signal averaging. During the experiments on the BioSpec 7 T scanner, hydrogen gas was enriched with para-isomer using a home-built setup operating at liquid nitrogen temperature, which delivers ca. 50% p-H_2_ fraction. For PHIP experiments, para-enriched hydrogen gas was bubbled into the sample solution through a 1/16″ (≈1.6 mm) O.D. PTFE capillary at a flow rate of 30 sccm at 2.8 bar for 20 s at 45 °C (unless specified otherwise). Hydrogenation reactions were carried out in the standard 5 mm NMR tubes equipped with wye-type fitting, filled with 600 μl of the substrate–catalyst solution. The details of the experiments are presented in Supplementary Table S1. NMR tube was placed inside the μ-metal shield with p-H_2_ pressure of 2.8–3 bar. Parahydrogen was bubbled through the solution at the low rate of 30 sccm for 20 s. After that, the sample was transferred from the low magnetic field to the Earth’s field (ca. 1 s), and then to the high magnetic field of the detection area. Further details on the MFC procedure are provided in the next section. In the experiments with INEPT-type pulse sequences, the hydrogenation reaction and the acquisition of spectra or images were carried out at the same high magnetic field. During the optimization of experimental conditions for NMR, multiple experiments were performed to ensure the reproducibility of the results. The reported signal enhancements are the mean values of five independent experimental results; the variations did not exceed 10%.

### ***The dependence of P(***^***13***^***C) on magnetic field in the MFC experiment***

To measure the magnetic field dependence of the PHIP transfer efficiency, we have used a 9.4 T Bruker Avance 400 NMR spectrometer equipped with an automated field cycling setup^44^. A gas bubbling system based on computer controlled TTL valves allowed us to perform repetitive bubbling with high reproducibility^62^. The sample was bubbled for 20 s in the magnetic shield placed on top of the spectrometer magnet. The magnetic field inside the shield was controlled by adjusting the current in the electromagnetic coils positioned inside it. After bubbling, the sample was transferred to the high magnetic field of the detection area, where ^13^C NMR spectrum was acquired. Before the sample transfer, a field jump of 25 μT was applied to make the field change non-adiabatic. The total delay between bubbling and detection was 1.2 s, including 0.6 s for sample transfer and 0.5 and 0.1 s waiting times with the sample in the shield and in the probe, respectively. The entire field dependence was measured with a single sample. The depletion of the substrate was taken into account by adding reference points measured for a predefined magnetic field (400 nT for vinyl acetate, 200 nT for propargyl pyruvate) in between every five points of the field dependence. This allowed us to assess the amount of PHIP produced in every measurement of the field dependence data points and to exclude the effect of chemical transformation of the substrate on the measured field dependence of signal enhancement. The temperature in the rf probe of the spectrometer was maintained at 45 °C. The parahydrogen bubbling pressure was set at 3 bar.

### Calculation of optimal delays for the PH-INEPT-PLUS sequence

The simulation was carried out for the spin density matrix of two protons in a singlet state and all other nuclei being non-polarized. All non-diagonal elements of the density matrix written in the high field eigenbasis were removed reflecting the coherence damping due to averaging over hydrogenation time. The resulting matrix was subjected to a set of rotations and free evolution delays corresponding to the PH-INEPT-PLUS pulse sequence. Transverse magnetization of ^13^C nuclei was calculated for various τ_1_ and τ_2_ delays to estimate their optimal values (Fig. 3b).

### MRI on a 9.4 T vertical bore Bruker Avance III spectrometer

MR images were acquired on a 9.4 T vertical bore Bruker Avance III spectrometer equipped with microimaging accessories using commercially available 25-mm (for [1-^13^C]acetate ester) and 30-mm (for [1-^13^C]pyruvate ester) ID ^13^C/^1^H RF coils. All MRI experiments were performed using gradient echo pulse sequence (fast low angle shot, FLASH)^63^ with one scan (no signal averaging). The total acquisition time was less than 1 s with the following parameters for FLASH MRI: repetition time (TR) of 3.1 ms, echo time (TE) of 1.5 ms, 16 phase encoding steps, 64 readout points, acquisition bandwidth 20 kHz, Cartesian spatial encoding. The durations of the phase encoding and readout gradients were 400 μs and 2.1 ms, respectively; the strength of both gradients was set at 6 G/cm. The FOV was 3.07 × 3.07 cm^2^. The initial 64 × 16 matrix was zero-filled to 64 × 64 for better presentation so that the digital in-plane resolution is similar in both dimensions. The resulting images were zoomed to eliminate the uninformative parts. As a result, ^13^C images were obtained with the spatial resolution of 0.48 × 1.92 mm^2^/pixel (and digital resolution of 0.48 × 0.48 mm^2^/pixel after zero-filling). During the experiments, the signal was collected from the entire volume of the sample. The flip angle was 15 degrees for [1-^13^C]acetate ester and 5 degrees for [1-^13^C]pyruvate ester. It was possible to reduce the flip angle for [1-^13^C]pyruvate ester because of the higher signal intensity.

### In vitro MRI on a Bruker BioSpec 7 T instrument

Experiments on a Bruker BioSpec 7 T imager equipped with a 40-mm ID ^13^C/^1^H RF volume coil were done in the following way: first, the pressurized sample was placed in a three-layer μ-metal shield where it was heated using a heat gun. In some experiments, the samples were additionally preheated before placing them in the shield (see Supplementary Table S1). The hydrogenation was achieved by bubbling parahydrogen through the solution with the sample residing in the shield. After the hydrogenation, the sample was de-pressurized and transferred to the animal imaging coil with the subsequent transfer to the scanner. The sample transfer time from the μ-metal shield into the Earth’s field took ca. 1 s. MRI experiments were done using RARE (rapid acquisition with relaxation enhancement) pulse sequence. For ethyl [1-^13^C]acetate, the slice thickness was 45 mm, rare factor 32, receiver gain 203, acquisition bandwidth 18 kHz, field of view 50 × 50 mm^2^, matrix size 32 × 32, the spatial resolution 1.56 × 1.56 mm^2^/pixel, TE = 61.8 ms, TR = 163 ms. For allyl [1-^13^C]pyruvate, the slice thickness was 10 mm, rare factor 32, receiver gain 64, acquisition bandwidth 18 kHz, field of view 54 × 50 mm^2^, matrix size 32 × 32, the spatial resolution 1.69 × 1.56 mm^2^/pixel, TE = 61.8 ms, TR = 163 ms.

## Supplementary Information

Supplementary Information
