# Supplementary material for: PHIP hyperpolarized [1-13C]pyruvate and [1-13C]acetate esters via PH-INEPT polarization transfer monitored by 13C NMR and MRI
Source: Sci Rep. 2021 Mar 11;11:5646. doi: 10.1038/s41598-021-85136-2 (PMC7952547; doi:10.1038/s41598-021-85136-2)
Supplement: Supplementary file 1 — Supplementary Information [file 41598_2021_85136_MOESM1_ESM.pdf]

PHIP hyperpolarized [1-^{13}C]pyruvate and [1-^{13}C]acetate esters via PH-INEPT polarization transfer monitored by ^{13}C NMR and MRI

Alexandra Svyatova,[1,2,3†] Vitaly P. Kozinenko,[1,2†] Nikita V. Chukanov,[1,2]
Dudari B. Burueva,[1,2] Eduard Y. Chekmenev,[4,5,6,7] Yu-Wen Chen,[8]
Dennis W. Hwang,[8]* Kirill V. Kovtunov,[1,2] and Igor V. Koptyug[1]*

[1]International Tomography Center SB RAS, 3A Institutskaya St., Novosibirsk, 630090, Russia
[2]Novosibirsk State University, 2 Pirogova St., Novosibirsk, 630090, Russia
[3]Institute of Cytology and Genetics SB RAS, 10 Ac. Lavrentieva Ave., Novosibirsk, 630090, Russia
[4]Department of Chemistry, Wayne State University, Detroit, MI 48201, USA
[5]Karmanos Cancer Institute, Wayne State University, Detroit, MI 48201, USA
[6]Integrative Biosciences, Wayne State University, Detroit, MI 48201, USA
[7]Russian Academy of Sciences, Moscow, 119991, Russia
[8]Institute of Biomedical Sciences, Academia Sinica, Taipei, 115 (Republic of China), Taiwan
*koptyug@tomo.nsc.ru, dwhwang@ibms.sinica.edu.tw
[†]These authors contributed equally to this work

Table of contents

Experimental details 2
Calculation of PHIP transfer field dependence in MFC experiments 3
^{13}C NMR/MRI on Bruker BioSpec 7 T of ethyl [1-^{13}C]acetate 4
^{13}C NMR/MRI on Bruker BioSpec 7 T of allyl [1-^{13}C]pyruvate 5
References 6

Experimental details

Vinyl acetate (VA) with ^{13}C at natural abundance was purchased from Sigma-Aldrich, propargyl pyruvate (PP) with ^{13}C at natural abundance, vinyl [1-^{13}C]acetate (1-^{13}C-VA) and propargyl [1-^{13}C]pyruvate (1-^{13}C-PP) were synthesized according to procedures reported elsewhere.[1]

Catalyst (1): [Rh(NBD)(dppb)]BF_4 (NBD = 2,5-norbornadiene, dppb = 1,4-bis(diphenylphosphino)-butane) prepared from commercially available [Rh$(NBD)_2$]BF_4 (Strem Chemicals, 96 %) and dppb ligand (Sigma-Aldrich, 98 %) in a 1:1 ratio.

Catalyst (2): commercially available [Rh(dppb)(COD)]BF_4 (COD = 1,5-cyclooctadiene) (Sigma-Aldrich, 98 %)

Table S1. The experimental details

	Substrate, concentration	Catalyst	Hydrogenation conditions	Polarization transfer approach
Figure 2b, Figure 2d	1-^{13}C-VA, 80 mM	Catalyst (1), 5 mM	45 °C<br>p-H_2 (85 %) was bubbled at 30 sccm, 2.8 bar, 20 s	MFC at 400 nT
Figure 2c	VA, 200 mM	Catalyst (2), 2.5 mM	45 °C<br>p-H_2 (85 %) was bubbled at 30 sccm, 3 bar, 20 s	MFC setup
Figure 3d, Figure 3e	1-^{13}C-VA, 80 mM	Catalyst (1), 5 mM	45 °C<br>p-H_2 (85 %) was bubbled at 30 sccm, 2.8 bar, 20 s	PH-INEPT-PLUS
Figure 4b, Figure 4d	1-^{13}C-PP, 80 mM	Catalyst (1), 5 mM	45 °C<br>p-H_2 (85 %) was bubbled at 30 sccm, 2.8 bar, 20 s	MFC at 200 nT
Figure 4c	PP, 200 mM	Catalyst (2), 2.5 mM	45 °C<br>p-H_2 (85 %) was bubbled at 30 sccm, 3 bar, 20 s	MFC setup
Figure 5d, Figure 5e	1-^{13}C-PP, 80 mM	Catalyst (1), 5 mM	45 °C<br>p-H_2 (85 %) was bubbled at 30 sccm, 2.8 bar, 20 s	PH-ECHO-INEPT-PLUS
Figure S2, Figure S3	1-^{13}C-VA, 0.8 M<br>1-^{13}C-PP, 0.18 M	Catalyst (1), 5 mM	All the experiments excluding MRI with 1-^{13}C-PP were done without preheating. The preheating of 1-^{13}C-PP was done using the heat gun.<br>p-H_2 (~50 %) was bubbled at 25 sccm, 2.8 bar, 15 s (in the experiments with 1-^{13}C-VA), 45 s (NMR with 1-^{13}C-PP) and 60 s (MRI with 1-^{13}C-PP)	MFC at ~0.1 μT

Calculation of PHIP transfer field dependence in MFC experiments

The numerical calculation of the field dependence was performed for a spin density matrix of two protons in a singlet state and all other nuclei being non-polarized. First, we omitted all non-diagonal elements of the density matrix written in the eigenbasis of the Hamiltonian at a specific magnetic field. This reflects the coherence averaging in the course of chemical reaction, which is valid in the case of PHIP. After that, the density matrix was projected on the high field eigenbasis and the expectation value of ^{13}C longitudinal magnetization (defined by S_z spin operator) was calculated. Such type of simulation corresponds to the case of instantaneous field switching. This assumption is correct in the described experiments, due to the field jump performed by an additional coil in the magnetic shield as described above. The list of J-couplings used for calculation is presented in Table S2, Table S3.

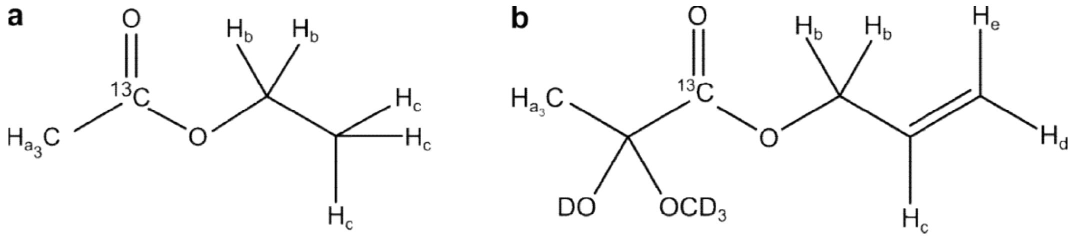

Figure S1. Notations for the nuclei of ethyl acetate (a) and allyl pyruvate (b) used in Table S2 and Table S3, respectively.

Table S2. J-coupling values in ethyl acetate used for calculations. All coupling constants below 1 Hz were taken from the literature.[2] All other constants were derived from spectra simulation performed using MestreNova software by comparing the simulated spectra with the experimentally obtained ones.

J, Hz	H_a	H_b	H_c	^{13}C
H_a		-	-	-7.0
H_b			7.2	3.1
H_c				-0.3
^{13}C				

Table S3. J-coupling values in allyl pyruvate used for calculations. All coupling constants below 1 Hz were taken from the literature.[2] All other constants were derived from spectra simulation performed using MestreNova software by comparing simulated spectra with the experimentally obtained one.

J, Hz	H_a	H_b	H_c	H_d	H_e	^{13}C
H_a		-	-	-	-	1.5
H_b			5.6	1.4	1.7	3.15
H_c				10.5	17.2	-0.18
H_d					1.4	0.20
H_e						0.08
^{13}C						

^{13}C NMR/MRI of ethyl [1-^{13}C]acetate on a Bruker BioSpec 7 T instrument

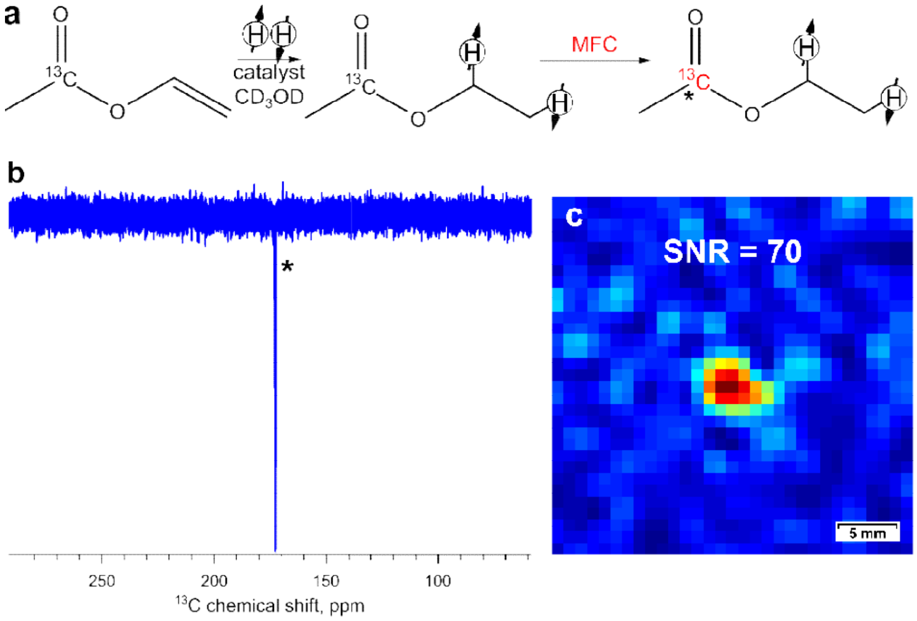

Figure S2. a) The reaction scheme of hydrogenation of vinyl [1-^{13}C]acetate. b) The ^{13}C NMR spectrum and (c) the ^{13}C MR image acquired after ^{13}C hyperpolarization of ethyl [1-^{13}C]acetate at 0.1 μT magnetic field. Slice thickness is 45 mm. The experiments were done using a Bruker BioSpec 7 T instrument.

^{13}C NMR/MRI of allyl [1-^{13}C]pyruvate on a Bruker BioSpec 7 T instrument

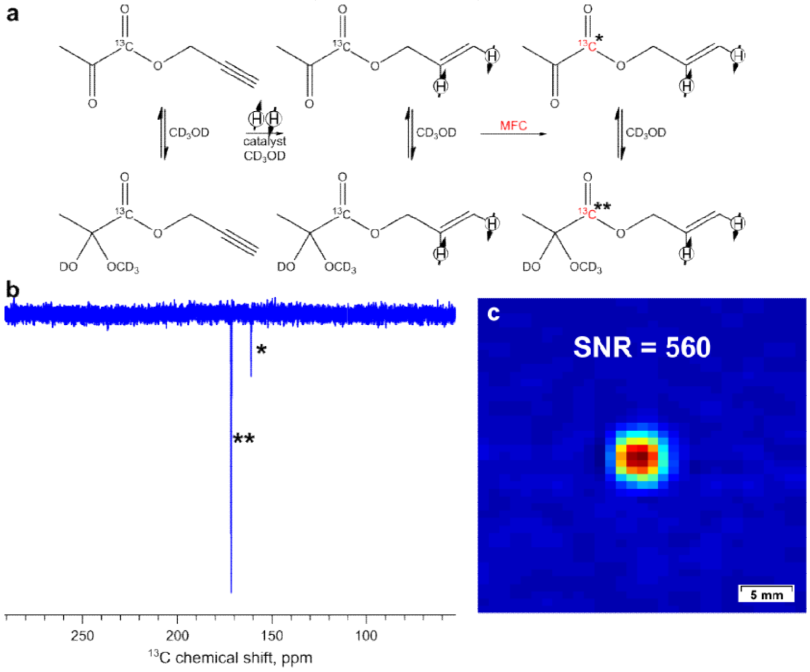

Figure S3. a) The reaction scheme of hydrogenation of allyl [1-^{13}C]pyruvate, b) The ^{13}C NMR spectrum and (c) the ^{13}C MR image acquired after ^{13}C hyperpolarization of allyl [1-^{13}C]pyruvate using MFC at 0.1 μT magnetic field. Slice thickness is 10 mm. The experiments were done using a Bruker BioSpec 7 T instrument.

References

1. Chukanov, N. V. *et al.* Synthesis of unsaturated precursors for parahydrogen-induced polarization and molecular imaging of 1-^{13}C-Acetates and 1-^{13}C-pyruvates via side arm hydrogenation. *ACS Omega* **3**, 6673–6682 (2018).

2. Stewart, N. J. *et al.* Long-range heteronuclear J-coupling constants in esters: Implications for ^{13}C metabolic MRI by side-arm parahydrogen-induced polarization. *J. Magn. Reson.* **296**, 85–92 (2018).
